# Discriminating Hepatocellular Carcinoma in Rats Using a High-T_c_ SQUID Detected Nuclear Resonance Spectrometer in a Magnetic Shielding Box

**DOI:** 10.1371/journal.pone.0047057

**Published:** 2012-10-05

**Authors:** Kai-Wen Huang, Hsin-Hsien Chen, Hong-Chang Yang, Herng-Er Horng, Shu-Hsien Liao, Shieh Yueh Yang, Jen-Jie Chieh, Li-Ming Wang

**Affiliations:** 1 Department of Surgery and Angiogenesis Center, National Taiwan University Hospital and National Taiwan University College of Medicine, Taipei, Taiwan; 2 Institute of Electro-optical Science and Technology, National Taiwan Normal University, Taipei, Taiwan; 3 Department of Electro-Optical Engineering, Kun Shan University, Tainan, Taiwan; 4 MagQu Co Ltd. Sindian Strict, New Taipei City, Taiwan; 5 Graduate Institute of Applied Physics and Department of Physics, National Taiwan University, Taipei, Taiwan; Mount Sinai School of Medicine, United States of America

## Abstract

In this study, we report the spin-lattice relaxation rate of hepatocellular carcinoma (HCC) and normal liver tissue in rats using a high-T_c_ superconducting quantum interference device (SQUID) based nuclear magnetic resonance (NMR) spectrometer. The resonance spectrometer used for discriminating liver tumors in rats via the difference in longitudinal relaxation time in low magnetic fields was set up in a compact and portable magnetic shielding box. The frequency-domain NMR signals of HCC tissues and normal liver tissues were analyzed to study their respective longitudinal relaxation rate T_1_^−1^. The T_1_^−1^ of liver tissues for ten normal rats and ten cancerous rats were investigated respectively. The averaged T_1_^−1^ value of normal liver tissue was (6.41±0.66) s^−1^, and the averaged T_1_^−1^ value of cancerous tissue was (3.38±0.15) s^−1^. The ratio of T_1_^−1^ for normal liver tissues and cancerous liver tissues of the rats investigated is estimated to be 1.9. Since this significant statistical difference, the T_1_^−1^ value can be used to distinguish the HCC tissues from normal liver tissues. This method of examining liver and tumor tissues has the advantages of being convenient, easy to operate, and stable.

## Introduction

Though sharing the same origin, there are still some morphologic distinctions that exist between normal and cancerous tissue. Nowadays, trained pathologists can discern the differences with certain kinds of immunohistochemical staining under microscopic examination. These are well-accepted tools for confirming the diagnosis of malignant diseases, and are also the main approach we employed to evaluate unknown specimens obtained from biopsy. Recently, a paper on the use of ultrasonography, computed tomography, and magnetic resonance imaging of hepatocellular carcinoma for improved treatment decision was reported [1]. While a different approach, the development of NMR measurement using SQUID detection in low magnetic fields [2]–[5], has been reported as well. The SQUID-detected magnetic resonance imaging shows promising applications for tumor detection as reported in [6], and has received much attention recently. Previously, we established a high-T_c_ SQUID-based low magnetic field nuclear magnetic resonance (NMR) system to measure the longitudinal relaxation rate T_1_^−1^ of tap water in a magnetically shielded environment [7], where T_1_^−1^ is the spin-lattice relaxation rate. While recently, we have established a SQUID-based NMR and MRI with radio frequency shielding and a high-T_c_ SQUID detector in superconducting shielding [8]. Since different tissues should show different T_1_^−1^. The T_1_^−1^ value is believed to also vary between normal and cancerous tissues. The goal of this research is to develop a novel diagnostic tool that is reliable, stable and more convenient than traditional methods in conducting pathological examinations with a resonance spectrometer set up in a compact and portable magnetic shielding box. Among miscellaneous cancers, hepatocellular carcinoma (HCC) is the most important malignancy in Asia [9], so we used HCC and normal liver parenchyma of rats as our first experiment subject to appraise the method’s feasibility.

## Materials and Methods

10 six-week-old male Wistar rats were chosen for the experiment. All rats were fed water mixed with 100 ppm of diethylnitrosamine for six weeks to induce HCC. All experiments were conducted with the approval of the Institutional Animal Care Committee. After six weeks, the rats’ livers developed tumors of 3–10 mm in diameter [10]. The rats were then euthanized via CO_2_ inhalation and the livers were harvested. Normal liver parenchyma and tumor tissues were stored separately in liquid nitrogen. Parts of the specimens were stored in formalin solution and hematoxylin, and then eosin staining was performed for prospective pathological studies. The pathologic diagnosis was independently made by two other pathologists to identify liver and tumor tissues.

In the past, the high-T_c_ SQUID-based NMR/MRI measurements were set up inside a magnetically shielded room [5]. Different from that system, the present resonance spectrometer was set up in a compact and portable magnetic shielding box. So far, it has been used to study the images of chili [9] and hyperpolarized ^3^He [11]. In this study, this NMR system was further optimized to discriminate tumors by characterizing the spin-lattice relaxation rate of HCC.

**Figure 1:**
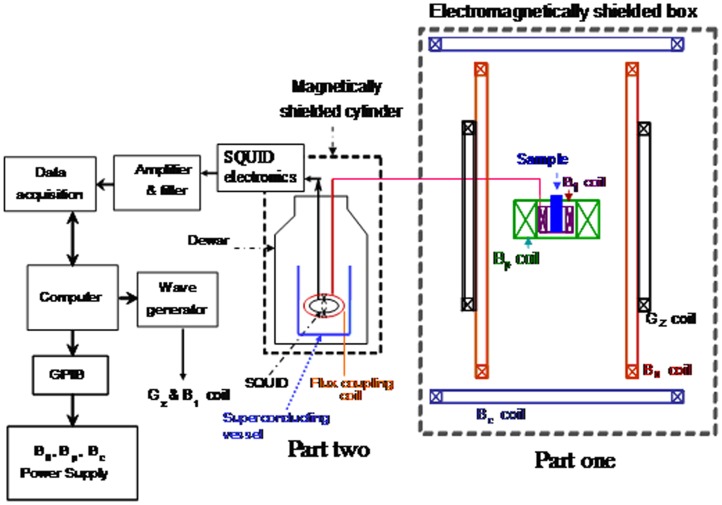
High-Tc SQUID-based NMR/MRI system.

**Figure 2:**
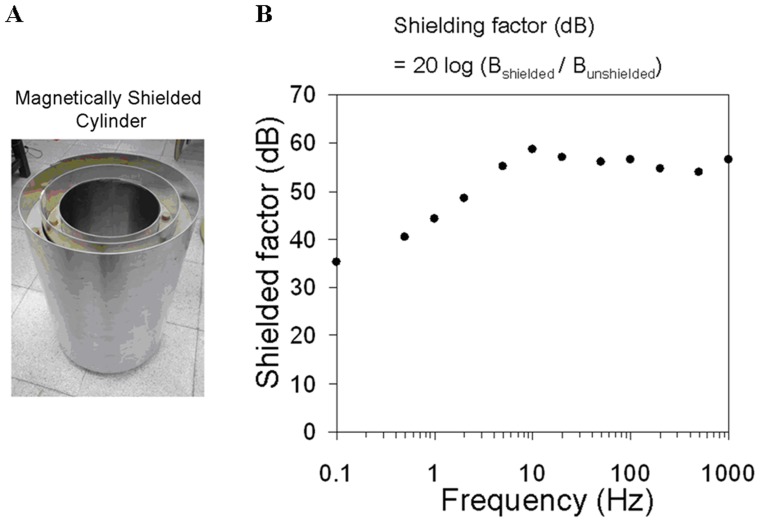
Magnetically shielded cylinder. (A) Photograph, and (B) shielding factor.

**Figure 3:**
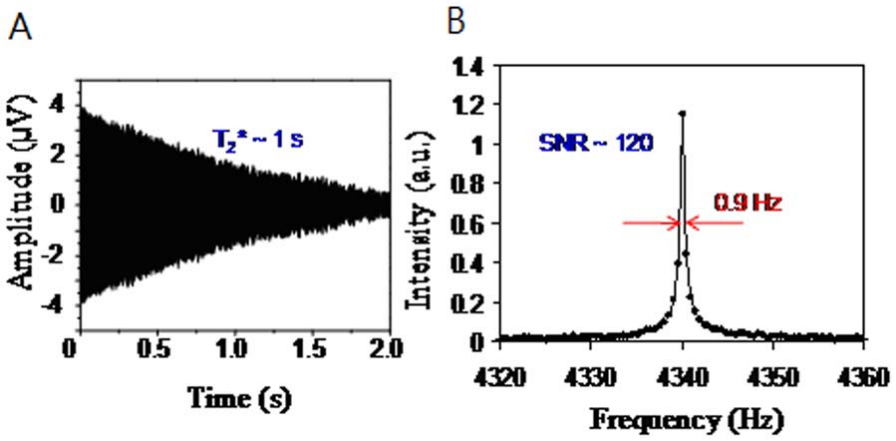
The test of 10 ml tap water. (A) NMR signal, and (B) frequency-domain NMR spectrum.

Figure 1 shows a schematic of the compact SQUID-detected resonance spectrometer. The system is consisted of two parts. Part one is consisted of the pre-polarization coil for generating B_p_, measuring coil for B_0_, pulse coil for B_1_, compensation coil for B_c_, and gradient coils for G_z_. These coils were setup inside an electromagnetically shielded box of four-layer aluminum. The vertical component of the Earth’s magnetic field, which was perpendicular to the measurement field, was compensated using the compensation coil. The stray gradient field was compensated using a gradient coil. The function of B_1_-coil is to generate a sinusoidal signal f(t) = A_1_sin(ωt), where A_1_ is the amplitude, ω is the resonance frequency which is equal to the Larmor frequency of protons. The pick-up coil inside part one and the input coil inside part two formed a tuned tank circuit, which resonates at the proton’s Larmor frequency with a Q-value of 20. The pick-up coil had an inner diameter of 6 mm, an inductance of ∼2.0 mH and was wound around the samples. The pick-up coil was placed inside the pre-polarization coil. The input coil had an inner diameter of 2.6 cm, coil length of 4 cm, inductance of ∼0.12 mH and was wound around the high-T_c_ SQUID detector. Part two is consisted of the high-T_c_ SQUID magnetometer, input coil, Bi_2_Sr_2_Ca_2_Cu_3_O_y_ vessel and magnetically shielded cylinder. The magnetically shielded cylinder (MSC) was made of three-layer mu-metal and aluminum plates. A photograph of the MSC is shown in Figure 2(a) and its shielding factor is shown in Figure 2(b). The shielding factor is defined as 20 log(B_shielded_/B_unshielded_), where B_shieded_ and B_unshielded_ are the magnetic field strength inside and outside the magnetically shielded box, respectively. The shielding factor of the MSC is 60 dB at 10 Hz and 55 dB at 1 k Hz. The high-T_c_ SQUID magnetometer and input coil were placed inside a superconducting vessel that was put in a liquid nitrogen dewar. The dewar was installed inside the mu-metal cylinder. The NMR signal of proton was inductively coupled to the SQUID magnetometer through a tuned tank circuit. Gradient coils were constructed for generating the gradient fields, G_x_, G_y_, and G_z_. After optimizing the NMR signals by using a high pre-polarization field and compensated gradient field, a signal-to-noise ratio of 120 for 10 ml of tap water was obtained and the frequency-domain spectral linewidth was 0.9 Hz in a single measurement as shown in Figure 3. This is comparable to the SQUID-based NMR system in a magnetically shielded environment [12]. The effective transverse relaxation time T_2_^*^ for the deionized water is T_2_^*^ = 1 s and the T_2_ = 2.2 s was derived from the spin-echo measurement at 24°C [13].

**Figure 4:**
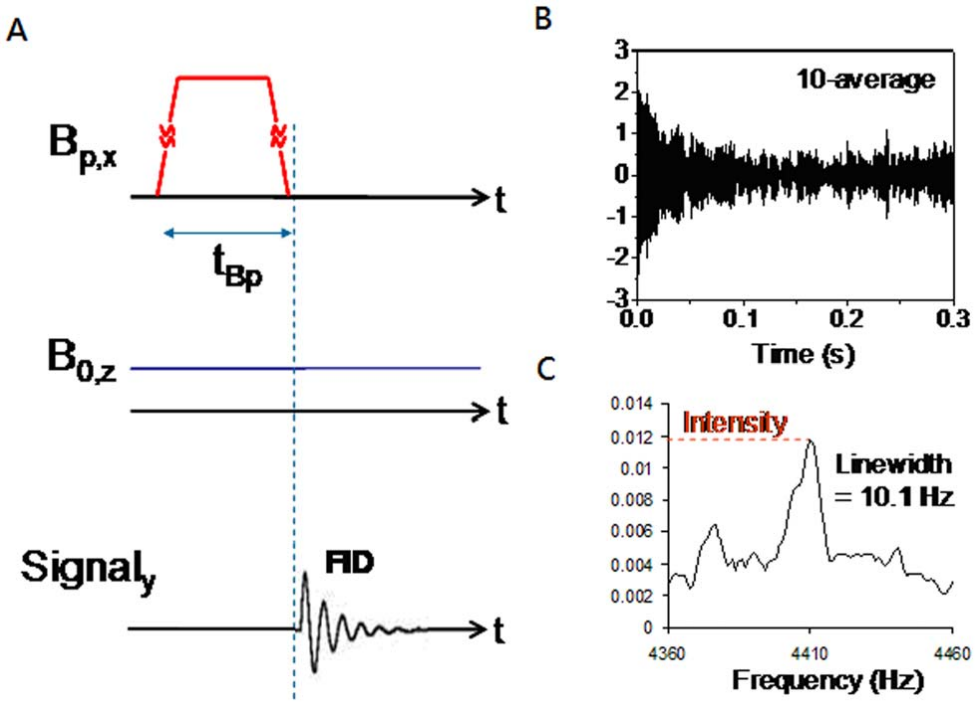
NMR tests of 1.3 g liver tissue of HCC rat. (A) Procedures applied for measuring the NMR signal of liver tissues, (B) FID of the NMR signal, and (C) frequency-domain NMR spectrum after 10 averages.

The procedures for T_1_ measurements are shown in Figure 4(a). In NMR measurements, the static field B_0_ of 100 µT was applied along the z-axis and the pre-polarization field B_p_ of 70 mT was applied along the x-axis. Since the pre-polarization field’s strength was much stronger than the static measuring field, the direction of the net nuclear spin magnetization of ^1^H aligned close to the x-axis. After applying a pre-polarization field for the time t_Bp_, the pre-polarization field was turned off in 3 ms. In the presence of the static field B_0_, the spin magnetization of ^1^H first processed inside the x-y plane and finally relaxed to the direction of z-axis. The free induction decay NMR signal was then recorded through the band pass filter and amplifier. As an example, the FID NMR signal of ^1^H nuclear spin for 1.3 gram of normal liver tissues in 10-average was recorded as shown in Figure 4(b). The frequency-domain NMR spectrum is shown in Figure 4(c), which shows a SNR of 3. The peak at 4.41 kHz is the resonance frequency of proton’s spins while the two side peaks are the 60 Hz harmonics.

To investigate the detection sensitivity of weight, both specimens of normal liver and HCC were divided into 5 pieces by weights that varied from 0.23 to 1.4 gram. The T_1_^−1^ value of each sample was measured in a triplet. Next, each liver and tumor sample were divided equally into four different groups with the same weight: Group A was stored at 300 K, Group B was frozen at the liquid nitrogen temperature (77.4 K) for examinations under a frozen status, Group C was also frozen in 77.4 K then defrosted to 300 K before any examinations were performed and frozen again after the experiment, while Group D was preserved in formalin solution. The T_1_^−1^ value of specimens in every group was obtained on the 1^st^, 2^nd^, 3^rd^, 4^th^, 5^th^, 6^th^, 7^th^, 30^th^, and 60^th^ day of experiments. In the end, each specimen was measured using a commonly used sample holder made from polyvinylchloride, glass, plastic and plastic wrap. The same evaluation was performed to see if the materials of sample holders would affect the T_1_^−1^ value or not.

## Results

**Figure 5:**
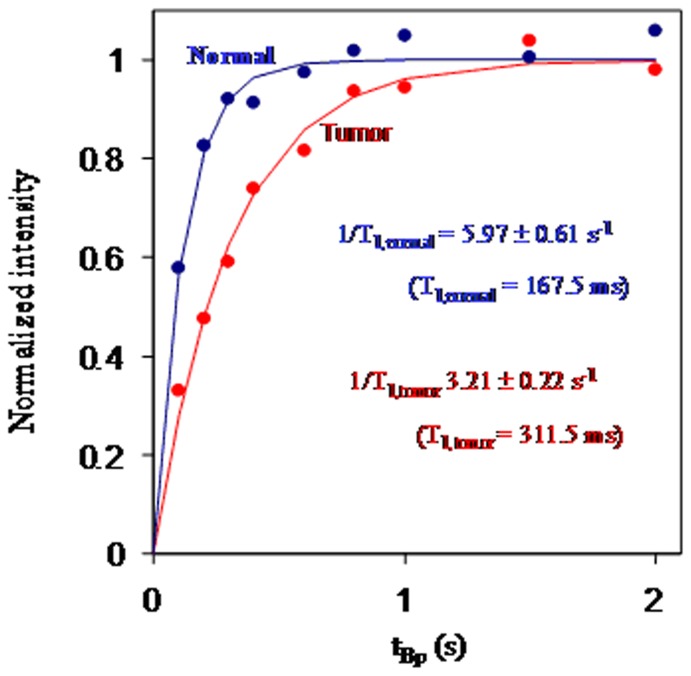
Normalized NMR intensity as a function of t_Bp_ for normal and tumor liver tissues.

Figure 5 shows the normalized intensity of NMR signals as a function of the pre-polarization time t_Bp_ for HCC tissues and normal liver tissues of rat. The data were normalized to the strength of saturated NMR signals to distinguish the signal characteristics for HCC tissues and normal liver tissues. The detected NMR signals S_y_(t) as a function of t_BP_ follows the formula:
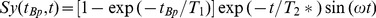
(1)where T_1_ is the longitudinal relaxation time, T_2_* is the effective transverse relaxation time and ω is the Larmor frequency of protons in the measuring magnetic field. The peak strength of frequency-domain NMR signal, as a function of t_Bp_, is fitted to [1- exp(−t_Bp_/T_1_)] to derive the T_1_ for tissues of HCC and normal livers. The dashed lines in Figure 5 are the fitting curves. 1/T_1,normal_ was equal to (5.96±0.61) s^−1^ (T_1,normal_ = 167.5 ms) and the 1/T_1,tumor_ was equal to (3.21±0.22) s^−1^ (T_1,tumor_ = 311.5 ms). The ratio (1/T_1,normal_)/(1/T_1,tumor_ ) is 1.86.

**Figure 6:**
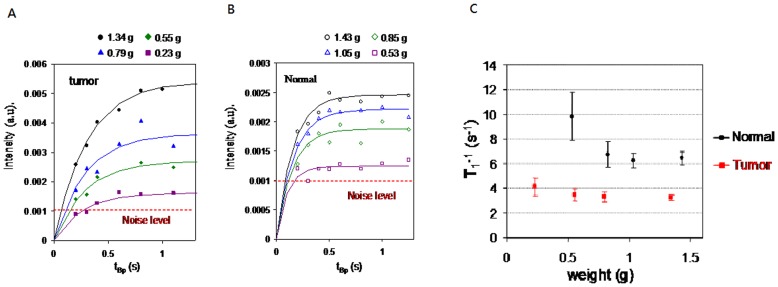
Intensity of NMR spectrum as a function of t_Bp_. (A) Different weight of the tumor in rat’s liver, (B) different weight of rat’s liver, and (C) T_1_^−1^ as a function of different weights of rat’s liver tissue and tumor tissue.

Figures 6(a) and 6(b) shows the normalized peak strength of the frequency-domain NMR signal as a function of t_Bp_ for different weights of the liver tumor and normal liver from the same rat. The solid lines are the fitting curves to [1- exp(−t_Bp_/T_1_)]. Figure 6(c) shows the derived T_1_’s for different weights of normal tissues and tumor tissues. The data of T_1_^−1^ under different weight conditions helped us come to several conclusions. First we found that the minimum specimen weight that can register a stable T_1_^−1^ value is 0.85 grams for normal liver tissue, and 0.23 grams for tumor tissue. Specimens with a weight greater than these values have a statistically stable T_1_^−1^ value. Therefore, 0.85 grams of liver tissues and 0.23 grams of tumor tissues are the minimum specimen weights that can be presently measured for the established system. The detection sensitivity of weight for cancerous tissues can be less than 0.23 grams if the system is further optimized by increasing the pre-polarization field’s strength and using a pickup coil in gradient configuration.

**Figure 7:**
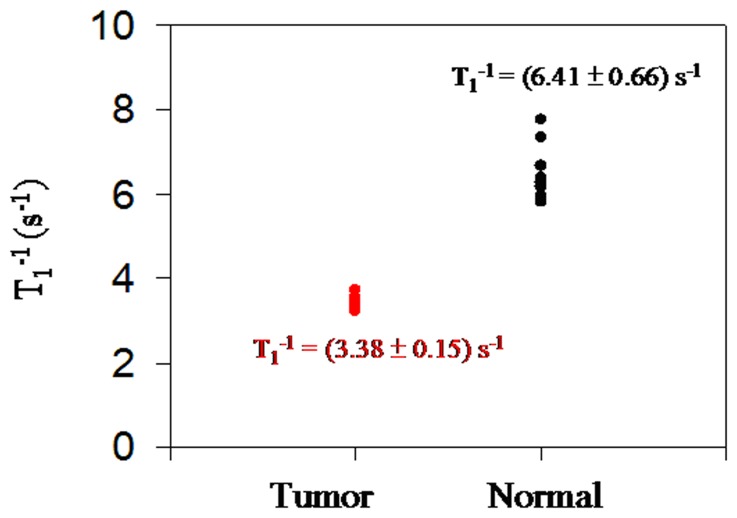
Averaged T_1_^−1^ of liver’s tissues. Samples are derived from ten normal rats and ten HCC rats.

Figure 7 shows the averaged T_1_^−1^ of liver’s tissues derived from 10 normal rats and 10 HCC rats. The averaged T_1_^−1^ value of normal liver tissue is (6.41±0.66) s^−1^, and the averaged T_1_^−1^ value of cancerous tissue is (3.38±0.15) s^−1^. The averaged T_1_^−1^ ratio between normal liver tissues and cancerous tissues is 1.9. The value suggests significant statistical difference between normal liver and cancerous tissues with p<0.05. The feasibility of using T_1_^−1^ values to discriminate cancerous liver tissues from normal liver is demonstrated. We note the experimental results of T_1_-relaxation in Figure 5 and 7 differ depending on the weight, storage time, portion of malignant tissues and environmental noises. The environmental noises can be improved by a pickup coil in gradient configuration and a high-T_c_ SQUID gradiometer [14]. The present system shows a minimum detection weight of 0.2 g. Increasing the weight of specimens to 0.6 g (see figure 6) can guarantee the accuracy of T_1_-relaxation measurements. Regarding the storage method, the experimental results show that it is highly recommended to store the samples in liquid nitrogen to preserve the T_1_-relaxation of tissues.

The water contents of normal and tumor tissues were studied in reference [15]. It was found that the data for malignant tissues show a clear trend towards increased water content. The observed difference between relaxation rates is attributed to greater tissue hydration in the neoplasms. In the present study, we demonstrate that (T_1_^−1^)_normal_>(T_1_^−1^)_tumor_ using a resonance spectrometer set up in a compact and portable magnetic shielding box.

**Figure 8:**
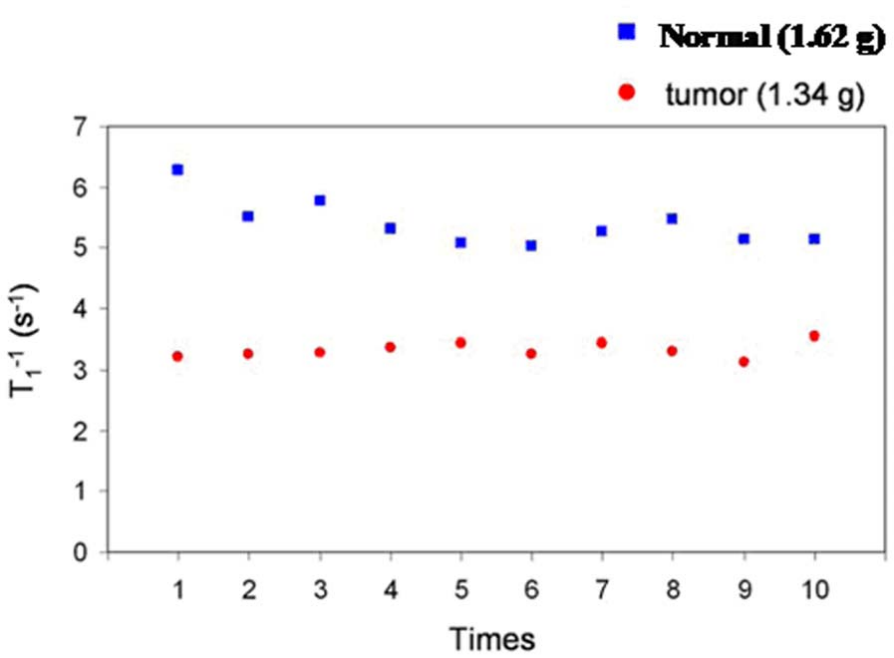
Ten repeated measurements of T_1_^−1^ at 300 K. Samples are rat’s normal liver tissue and tumor tissue when they are thermally cycled from liquid nitrogen.

**Figure 9:**
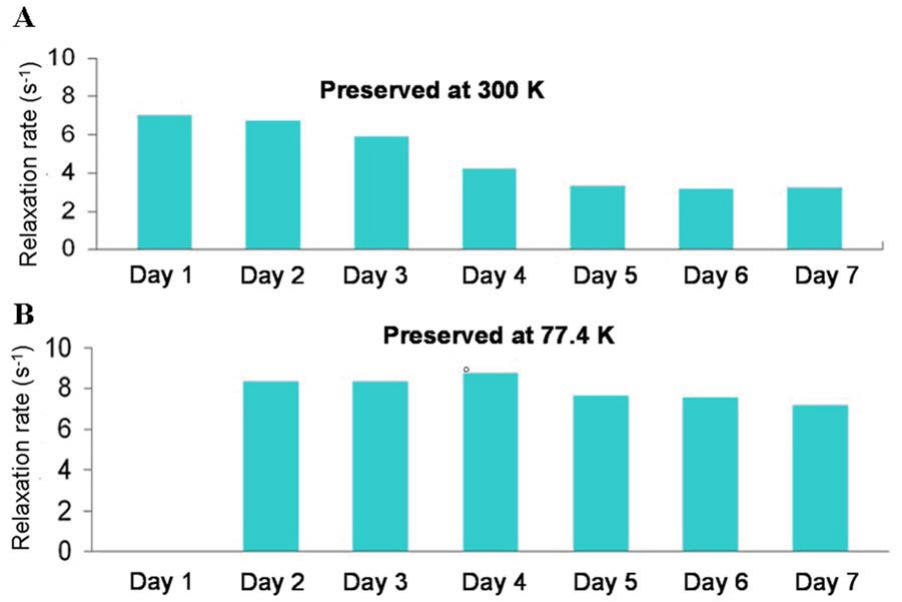
Repeated T_1_^−1^ measurements for rat’s liver tissue. It is kept at (A) 300 K, and (B) 77.4 K and recycled to 300 K.

Since tumor and normal samples are usually preserved in liquid nitrogen or formalin. It was interesting to investigate how many times we could recycle the samples without destroying the T_1_^−1^ characteristics. Figure 8 shows ten repeated T_1_^−1^ measurements at 300 K for a rat’s liver tissue and tumor tissue when they are thermally cycled from a liquid nitrogen environment. The total time including the warming up time (30 minutes) and the measurement time (15 minutes) in each T_1_^−1^ measurement was 45 minutes. The results show that the T_1_^−1^ value does not vary significantly and therefore the specimen can be stored at 77.4 K for a long period of time without deteriorating the T_1_^−1^. However, if the fresh specimen was not preserved and was kept at 300 K, the T_1_^−1^ value would continue to decrease as time proceeded, as shown in Figure 9(a). The T_1_^−1^ decreases significantly after exposure to air for two days while the liver tissues degrade. Samples preserved at 77.4 K and recycled to 300 for T1-measurement show no degrading after several days shown in figure 9(b). On the other hand, for the specimen that was preserved in formalin, the most common method of storing specimens, under the aforementioned conditions, we still were able to obtain consistent T_1_^−1^ values for sixty testing days.

The T_1_^−1^ of normal liver tissues and HCC tissue was also characterized using different specimen holders consisted of non-magnetic materials such as polyvinylchloride, plastics, plastic wrap, and glass. The results yield no statistical differences in T_1_^−1^ values due to the short relaxation rate of sample holders compared with that of the liver tissues. Therefore, we conclude that the T_1_^−1^ value is not influenced by nonmagnetic materials that are generally used to hold the specimen.

## Discussion

Currently, the main method of distinguishing normal liver tissues from HCC tissue depends on the pathological analysis of specimen obtained from biopsy. Such examination demands high human resource as this requires professional pathologists and it takes time. Moreover, when the quantity of specimen is undersize, there is usually an insufficient amount to conduct all the examinations. Therefore, the same small piece of specimen may need to be used for regular microscopic examination under the hematoxylin and eosin staining as well as many other immunohistochemical staining simultaneously for coming to a diagnosis. Furthermore, sometimes the specimens are depleted and unable to be used in follow-up pathological examinations. One characteristic of the method described here is that the specimen is not damaged. It can be used for other pathological analysis. This can provide more possibilities when examining undersized specimens.

In the present work, a resonance spectrometer set up in a compact and portable magnetic shielding box was optimized to discriminate tumor tissues from normal liver tissues. The averaged T_1_^−1^ value of normal liver tissue is characterized to be (6.41±0.66) s^−1^, and the averaged T_1_^−1^ value of cancerous tissue is (3.38±0.15) s^−1^. The (T_1,normal_^−1^)/(T_1,tumor_^−1^) was characterized to be 1.9, which is consistent with the previous results [16] measured in a magnetically shielded environment. Additionally, the tissues simply need to be stored in liquid nitrogen or preserved in formalin to maintain their efficacy. The present system is compact, the measurement is easy, and the system cost is lower. Therefore, it can also be a promising tool for characterizing different tumors by the parameter T_1_^−1^.

### Conclusions

In this study, we conclude that in 295 K, a stable T_1_^−1^ value can be measured for liver tissues of certain sizes without introducing irrelevant effects from the materials of containers. In addition, the tissues simply need to be stored in liquid nitrogen or preserved in formalin to maintain their efficacy. This method of examining and distinguishing liver and tumor tissues displays the advantages of being convenient, easy to operate, stable, and other benefits. Finally and most importantly, the noticeable difference in the T_1_^−1^ value between liver and tumor tissues provides a new direction of study in liver cancer pathology. This warrants further investigation of whether the T_1_^−1^ value of tumor tissues can be used to further distinguish various types of tumors.
