# Correction: Discriminating Hepatocellular Carcinoma in Rats Using a High-T_c_ SQUID Detected Nuclear Resonance Spectrometer in a Magnetic Shielding Box

**DOI:** 10.1371/annotation/f0b887f3-4636-4f29-ab81-a169247a3ad5

**Published:** 2014-01-03

**Authors:** Kai-Wen Huang, Hsin-Hsien Chen, Hong-Chang Yang, Herng-Er Horng, Shu-Hsien Liao, Shieh Yueh Yang, Jen-Jie Chieh, Li-Ming Wang

There was an error in the affiliations of the first author. Kai-Wen Huang’s affiliations are:

Department of Surgery & Hepatitis Research Center, National Taiwan University Hospital, Taipei, Taiwan, and

Graduate Institute of Clinical Medicine, College of Medicine, National Taiwan University, Taipei, Taiwan 

